# Charge Engineering Reveals the Roles of Ionizable Side Chains in Electrospray Ionization Mass Spectrometry

**DOI:** 10.1021/jacsau.1c00458

**Published:** 2021-11-29

**Authors:** Mia L. Abramsson, Cagla Sahin, Jonathan T. S. Hopper, Rui M. M. Branca, Jens Danielsson, Mingming Xu, Shane A. Chandler, Nicklas Österlund, Leopold L. Ilag, Axel Leppert, Joana Costeira-Paulo, Lisa Lang, Kaare Teilum, Arthur Laganowsky, Justin L. P. Benesch, Mikael Oliveberg, Carol V. Robinson, Erik G. Marklund, Timothy M. Allison, Jakob R. Winther, Michael Landreh

**Affiliations:** † Department of Microbiology, Tumor and Cell Biology, Karolinska Institutet, Tomtebodavägen 23A, 171 65 Stockholm, Sweden; ‡ Linderstrøm-Lang Centre for Protein Science, Department of Biology, University of Copenhagen, Ole Maaløes vej 5, 2200 Copenhagen, Denmark; § Department of Chemistry, University of Oxford, South Parks Road, Oxford OX1 3QZ, U.K.; ∥ Department of Oncology-Pathology, Science for Life Laboratory and Karolinska Institutet, 171 65 Stockholm, Sweden; ⊥ Department of Biochemistry and Biophysics, Stockholm University, 106 91 Stockholm, Sweden; # Department of Material and Environmental Chemistry, Stockholm University, 106 91 Stockholm, Sweden; ¶ Department of Biosciences and Nutrition, Karolinska Institutet, Neo, 141 83 Huddinge, Sweden; □ Department of Chemistry−BMC, Uppsala University, Box 576, 751 23 Uppsala, Sweden; ○ Department of Chemistry, Texas A&M University, College Station, Texas 77843, United States; △ Biomolecular Interaction Centre, School of Physical and Chemical Sciences, University of Canterbury, Christchurch 8140, New Zealand

**Keywords:** protein folding, gas-phase conformations, ion mobility mass spectrometry

## Abstract

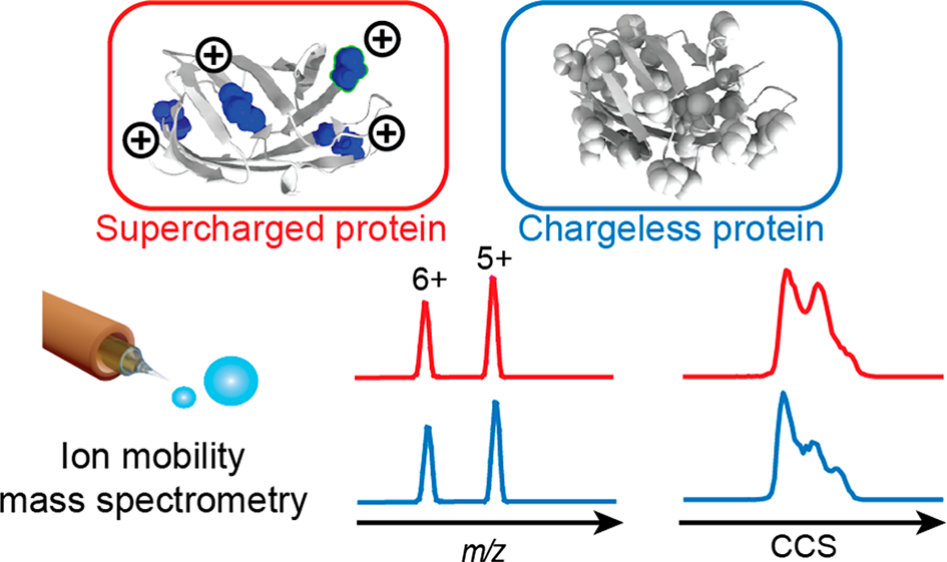

In solution, the charge of a protein is intricately linked to its stability, but electrospray ionization distorts this connection, potentially limiting the ability of native mass spectrometry to inform about protein structure and dynamics. How the behavior of intact proteins in the gas phase depends on the presence and distribution of ionizable surface residues has been difficult to answer because multiple chargeable sites are present in virtually all proteins. Turning to protein engineering, we show that ionizable side chains are completely dispensable for charging under native conditions, but if present, they are preferential protonation sites. The absence of ionizable side chains results in identical charge state distributions under native-like and denaturing conditions, while coexisting conformers can be distinguished using ion mobility separation. An excess of ionizable side chains, on the other hand, effectively modulates protein ion stability. In fact, moving a single ionizable group can dramatically alter the gas-phase conformation of a protein ion. We conclude that although the *sum* of the charges is governed solely by Coulombic terms, their *locations* affect the stability of the protein in the gas phase.

Characterizing the interactions, stabilities, and conformations of proteins is of central importance in biochemical and pharmaceutical sciences. Native mass spectrometry (MS) informs about the molecular weight of protein complexes to reveal oligomeric states and ligand binding.^1−3^ Combined with ion mobility (IM), it can be used to determine collision cross sections (CCS) and identify conformational changes.^4−6^ If protein complexes are additionally subjected to collisional activation inside the mass spectrometer, the resulting collision-induced unfolding (CIU) can be followed by IM-MS, informing about their gas-phase stabilities.^7−10^

IM-MS has been successfully applied to characterize the lipid preferences of membrane proteins,^11,12^ define structural heterogeneity associated with glycosylation,^13^ follow structural transitions in disordered proteins,^14^ and determine the impact of disease-associated mutations on protein stability.^15^ By combining alanine scanning with CIU, it is possible to quantify the contributions of individual residues on the conformational stabilities of protein ions.^16^ IM-MS is furthermore employed in the pharmaceutical industry,^17^ for example to characterize drug conjugates.^18^

Common to all of these applications is a reliance on the generation of native-like protein ions through electrospray ionization (ESI), where the protein is desolvated and charges are added to facilitate its detection in the mass spectrometer. A significant body of work over many decades has been dedicated to understanding the mechanism of protein charging in ESI and protein structures in the gas phase.^19−23^ However, while the three-dimensional structures of protein complexes can be preserved for the time frame of an MS experiment, their surface charges inevitably differ between solution and gas phase.^20,24,25^ In positive-mode ESI, the number of charges acquired by native proteins scales with their solvent-accessible surface area (SASA), as predicted by the charge residue model (CRM), and is in virtually all cases lower than the number of basic surface residues.^26−28^ Protein stability in solution depends on a delicate balance of surface electrostatics.^29^ Therefore, the charging process can distort protein stability by adding Coulombic repulsion between ESI charges.^30−32^ This discrepancy raises the question to what extent the surface properties of a protein can affect its behavior in the gas phase and thus in IM-MS. For example, drug conjugation of antibodies can alter their surface charges, and membrane proteins have a lower percentage and a more uneven distribution of ionizable residues on their surface than soluble proteins.^33^ Here, we clarify the role of ionizable residues in native MS using engineered proteins where the number of ionizable side chains can be altered without affecting their native structures.

## Results

### Ionizable Groups Connect Charging and Folding in Native MS

**Figure 1:**
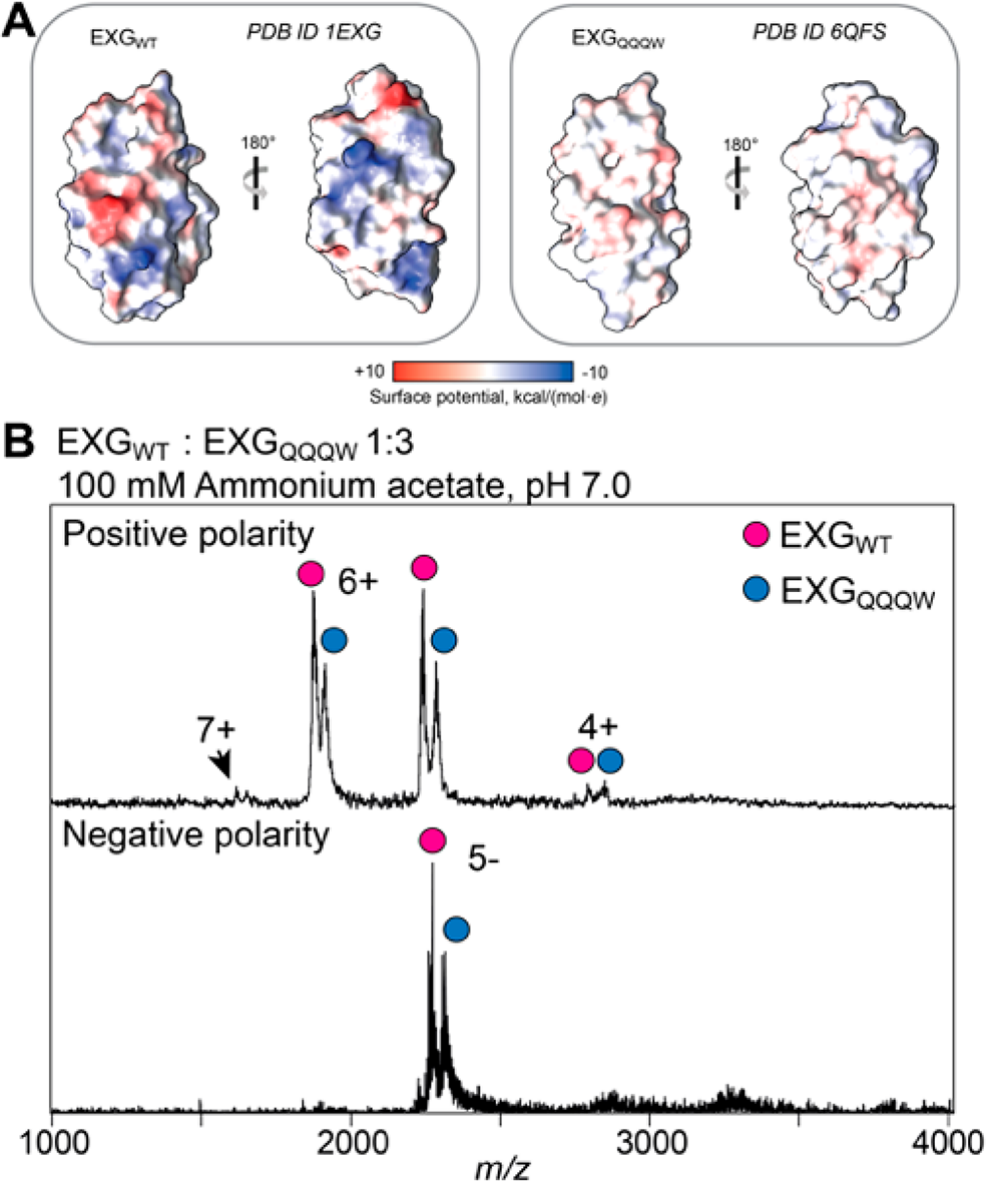
Charging of EXG variants in ESI-MS. (A) Wt EXG_WT_ and the chargeless variant EXG_QQQW_ have highly similar three-dimensional structures. Protein surfaces are rendered according to Coulombic potential at pH 7.0, showing the effect of four replacements (K28Q, D36Q, R68Q, and H90W) on the surface charge of EXG_QQQW_. (B) ESI-Mass spectra of a 1:3 mixture of EXG_WT_ and EXG_QQQW_ show identical charge state envelopes for both variants in positive (top) and negative (bottom) ionization modes.

First, to fully uncouple solution- and gas-phase charge, we turned to a cellulose-binding domain derived from an exoglycanase from *Cellulomonas fimi*. The wild-type protein has a low charge density, with three basic and one acidic residue in its 106-residue sequence (EXG_WT_) and includes one disulfide bridge. Replacement with uncharged residues (K28Q, D36Q, R68Q, and H90W), plus an N-terminal acetylation (EXG_QQQW_), has yielded a protein in which the C-terminus is the only ionizable site, while maintaining a near-identical structure as the wild-type (Figure 1A, Figure S1).^34,35^ ESI-MS of EXG_QQQW_ revealed two populations with masses of 11 246 and 11 290 Da instead of the expected 11 158 Da. Enzymatic digestion and LC-MS/MS analysis did not show any unexpected sequence variations or modifications. Collisional activation of the protein in the ion trap of the mass spectrometer resulted in a reduction of ion mass, revealing the expected molecular weight with 0–8 sodium adducts (Figure S2A). ESI-MS in negative mode similarly showed up to eight chloride adducts (Figure S2B). EXG_WT_, on the other hand, yielded the expected mass of 11 080 Da, with only minor adduct formation (Figure S2C). The presence of 0–8 sodium adducts for the EXG_QQQW_ 6+ ion suggests that they are not the sole charge carriers and could bind to the backbone or side chain carbonyls.^36,37^ EXG_QQQW_ may also retain NH_4_^+^, as shown previously for proteins with disproportionately few basic residues,^38^ which can be dissociated by collisional activation to reveal the more stable Na adducts. Such a “mixed charging” scenario can also involve protonation of side chains such as P and Q.^39^

We recorded ESI-mass spectra of a mixture of EXG_WT_ and EXG_QQQW_ in 100 mM ammonium acetate (AmAc), pH 7.0, in positive and negative ionization modes. We found that we required ∼3 times the concentration of EXG_QQQW_ to obtain a signal intensity comparable to that of EXG_WT_ (Figure 1B). Both proteins exhibited virtually identical narrow charge state distributions (CSDs). In positive mode, we found a maximum charge (*z*_max_) of 7+, just below the 8+ charge predicted by the SASA. Furthermore, the average charge of 5.4+ is in good agreement with the value of 5.5+ predicted based on the SASA of the crystal structure (Table 1).^38^ These findings clearly illustrate that ESI charging is independent of surface properties, but rather reflects key features of protein solution structure. This interpretation is in good agreement with reports that chemical modifications of side-chain charges do not affect ESI charges of compactly folded protein ions.^28,40^ To understand further the role of ionizable residues, we tested whether we could reduce the charge of EXG_WT_ and EXG_QQQW_ ions in the gas phase. Trimethylamine-*N*-oxide (TMAO) is a basic osmolyte that strips protons off ionized proteins through collisions in the gas phase.^41,42^ After addition of 50 mM TMAO to the ESI solution, both proteins shift to lower charge states (Figure S3). However, we also observed that the intensity of the EXG_QQQW_ peaks was significantly reduced. To explore the origin of this phenomenon, we increased the charge-reducing effect of TMAO through increased collisional activation in the ion source. At a cone voltage of 300 V, the average charge of the EXG_WT_ was reduced to 2.7, while the EXG_QQQW_ signal was lost. We conclude that a subset of charges on EXG_WT_ are bound to high-affinity sites that render them resistant to stripping by TMAO, whereas EXG_QQQW_ can be decharged completely and is lost as a neutral species. In summary, although both variants acquire the same number of charges, the presence of basic residues in EXG_WT_ results in a higher gas phase basicity (GB).

**Table 1:** SASAs, Predicted and Experimental Maximum Charge (*z*_max_), and Predicted and Experimental Average Charge (*z*_avg_) for All Protein Variants

protein	SASA (Å^2^)	pred. *z*_max_	exp. *z*_max_	pred. *z*_avg_	exp. *z*_avg_
EXG_WT_	5080	8.2	7	5.5	5.5
EXG_QQQW_	4594	8.3	7	5.4	5.4
GFP_WT_	11 170	13.1	12	9.7	9.7
GFP_Ac_	9941	13.1	11	9.2	9.2
GFP_Bas_	11 601	13.2	12	10.1	10.1
TTHA	4273	6.5	5	5.2	4.8

### Protein Folded States Can Be Distinguished in the Absence of Ionizable Residues

**Figure 2:**
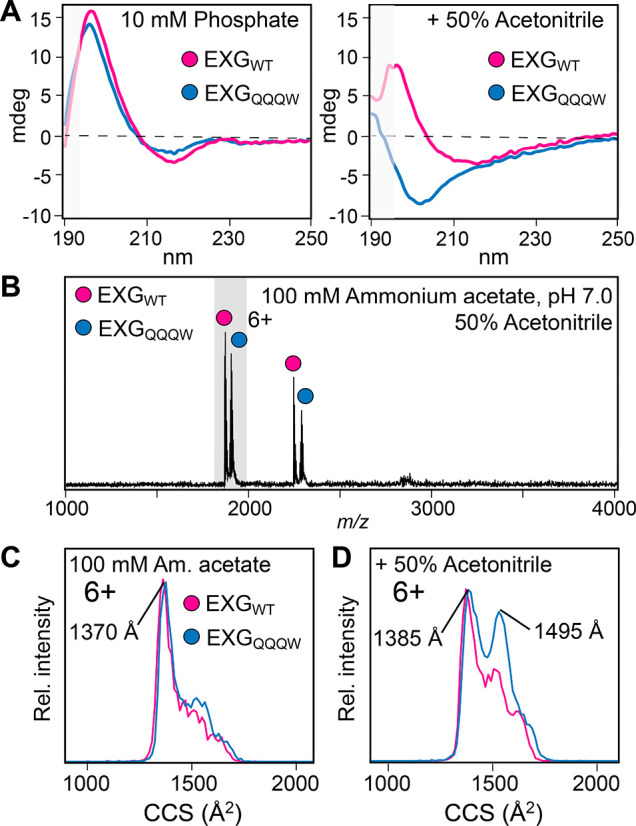
Folded states of EXG_WT_ and EXG_QQQW_ in solution and in the gas phase. (A) CD spectroscopy in the far-UV region shows near-identical secondary structures in 10 mM sodium phosphate buffer pH 7. In 50% acetonitrile, EXG_QQQW_, but not EXG_WT_, converts to random coil. Greyed-out parts of the curves exhibited HT voltages above 700 V, indicating increased spectral noise. (B) ESI-MS shows a slight increase of the average charge to 5.7 for both proteins sprayed from 50% acetonitrile. (C) In 100 mM AmAc, the 6+ charge states of EXG_WT_ and EXG_QQQW_ have similar CCS distributions. (D) In 50% acetonitrile, the CCS distribution for EXG_QQQW_ shows an extra peak around 1550 Å^2^, indicating a second, more unfolded population.

Because both proteins exhibited identical charge states under all conditions, we asked whether we could still distinguish coexisting conformational states by MS. Using circular dichroism (CD) spectroscopy, we found that the addition of the common denaturant acetonitrile to the buffer (50% v/v) converted EXG_QQQW_ to random coil, whereas EXG_WT_ appears to retain more of its native secondary structure (Figure 2A). MS analysis of the proteins in 50% acetonitrile showed identical CSDs with only a minor shift to higher charge states compared to native conditions (Figure 2B). Despite unfolding in solution, the charge states of EXG_WT_ and EXG_QQQW_ again remain close to the limit imposed by the SASA of the native protein.

Next, we employed IM-MS, where protein ions are separated according to mobility, which depends on their charges and their CCSs.^5^ In this manner, we can distinguish conformational ensembles with identical mass-to-charge ratios. Analysis of the main 6+ charge state under native conditions revealed identical CCS distributions for EXG_WT_ and EXG_QQQW_, with a narrow peak at 1370 Å^2^ and a shoulder around 1500 Å^2^ (Figure 2C). Comparison to the expected value of 1240 Å^2^ theoretically calculated from the crystal structure suggests a compact conformation in the gas phase. The small increase over the predicted CCS may be due to the disordered N-terminus, as well as some Coulombic stretching due to the high charge. Consistent with this interpretation, the CCS determined for the 5+ charge state is centered at a peak of 1280 Å^2^ and exhibits a good agreement with the theoretical value (Figure S4). We then investigated the effect of 50% acetonitrile on the CCS distributions of both proteins. For the 6+ charge state of EXG_WT_, we found a small increase in the unfolded fraction with a CCS of around 1500 Å^2^, while most of the protein retained the same CCS as under native conditions. For the 6+ ions of the chargeless variant, the intensity of the CCS distribution around 1500 Å^2^ increased notably, indicating a nearly 1:1 ratio of compact to unfolded protein (Figure 2D). It appears therefore that despite their identical CSDs, an increased population of EXG_QQQW_ variant occupies an unfolded state, reflecting the solution behavior observed by CD spectroscopy.

CIU was then employed to test whether ionizable residues affect the folding landscape of EXG in the gas phase. Briefly, protein ions are subjected to thermal activation in the mass spectrometer by raising the acceleration voltage of ions entering the collision cell, leading to an increase in high-energy collisions with the neutral buffer gas. The resulting unfolding events can be monitored by following the change in CCS as a function of collision energy.^7^ Surprisingly, the CIU traces obtained for EXG_WT_ and EXG_QQQW_ showed no significant differences in stability, with both reaching an unfolded state at 15 V (Figure S5). We reason that for the same charge state, the gas-phase stabilities of both variants are governed mainly by the energy required to disrupt their identical or highly similar internal hydrogen-bonding networks.

### Surface Charge Distributions Control the Stability of Protein Ions

**Figure 3:**
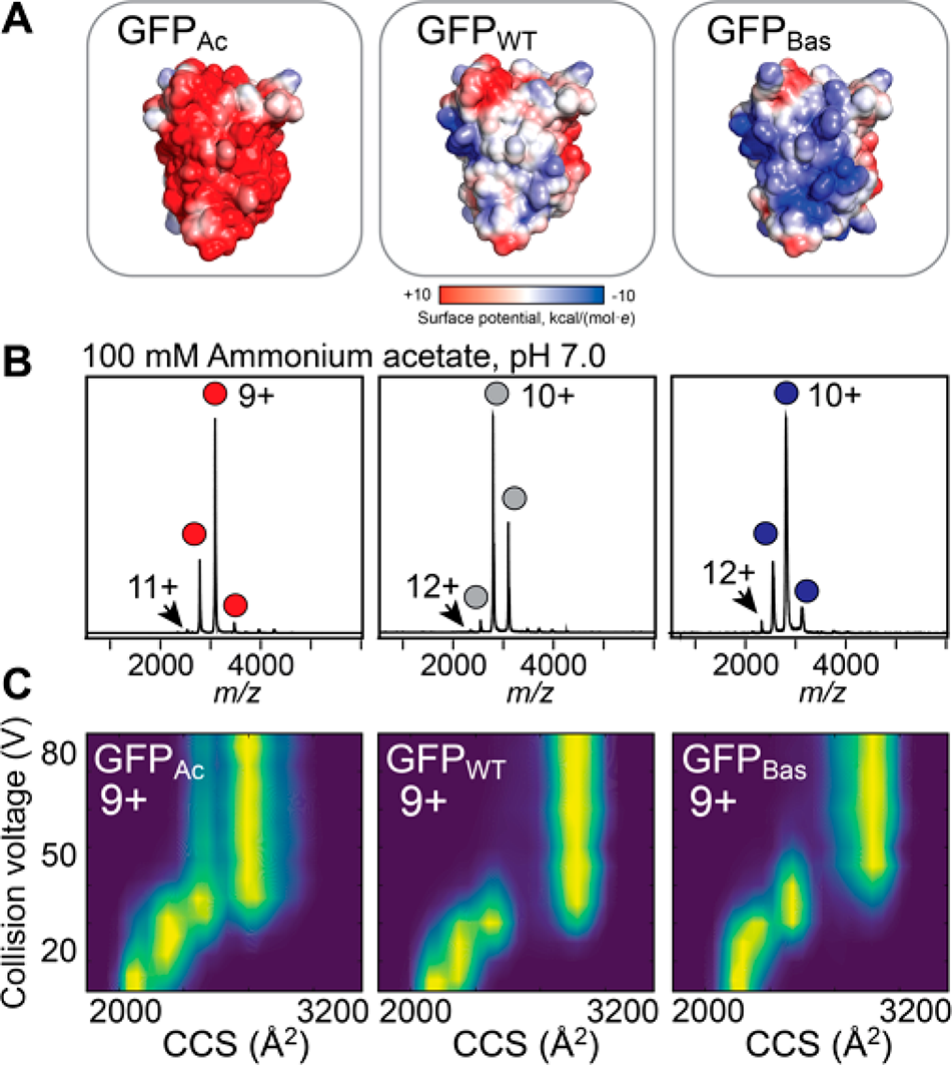
Charging and gas-phase stabilities of WT and supercharged GFP variants. (A) Coulombic surface potentials predicted for the three GFP variants. Structural models were generated using Rosetta; the His_6_ tag is omitted for clarity. (B) Mass spectra of GFP_Ac_, GFP_WT_, and GFP_Bas_ show near-identical CSDs dominated by the 9+ and 10+ charge states. (C) CIU profiles of the 9+ charge state of each variant indicate that GFP_Bas_ has a higher resistance to unfolding than GFP_WT_ and GFP_Ac_.

Having established that charge depletion does not necessarily change gas-phase unfolding, we asked whether we could modulate the stability of protein ions by instead adding ionizable sites. For this analysis, we utilized two green fluorescent protein (GFP) variants that maintain fluorescence and have near-identical secondary and tertiary structure to the wild-type (GFP_WT_) protein (28 basic and 34 acidic residues), in which a large number of surface-exposed residues have been mutated to have either acidic (GFP_Ac_, 19 basic and 49 acidic residues) or basic (GFP_Bas_, 41 basic and 26 acidic residues) side chains (Figure 3A).^43^ Varying only in the charge of the surface-exposed amino acids, this system is therefore ideal to study how an excess of ionizable side chains affects protein ion stability. As expected, mass spectra of GFP_WT_, GFP_Ac_, and GFP_Bas_ exhibit near-identical CSDs with average charge states of 9.7+, 9.2+, and 10.1+, respectively (Figure 3B, Table 1), reflecting their highly similar SASAs. IM-MS analysis yield CCSs of approximately 2000–2100 Å^2^ for all three variants, in good agreement with the 2118 Å^2^ predicted from the GFP_WT_ structure. However, the CIU profiles of the 9+ and 8+ charge states of GFP_WT_ and the basic and acidic variants reveal different gas-phase stabilities, as well as a smaller CCS for the unfolded state of GFP_Ac_ (Figure 3C, Figure S6). To assess the difference in stability, we performed CIU of all GFP variants. By analyzing the proteins pairwise, we could quantify the stabilities of the most compact state of each variant relative to the GFP_WT_.^10^ The wild type and acidic variant unfold at low collision voltages, whereas the basic variant is more stable (Figure S7). Interestingly, solution unfolding analysis has not revealed significant differences in stability of supercharged GFP variants.^43^ Because all three variants share the same three-dimensional fold and hydrophobic core, the data show that despite making no notable contribution to ion charge, increasing the number of basic residues on the protein surface can have a significant impact on the gas phase stabilities of positively charged protein ions.

We thus asked if the increased stability of GFP_Bas_ could be related to the distribution of the ESI charges. We devised a Monte Carlo (MC) scheme for transferring protons (See Supporting Information) where we omit the dynamics of the overall structure and simplify the otherwise slow rearrangement of side chains in vacuum,^44,45^ thus getting a more exhaustive sampling compared to hybrid MC-MD approaches^46,47^ while also removing any bias from the surface details in the atomistic structure used as input. Briefly, we used the GFP_WT_ crystal structure and Rosetta structures for GFP_Ac_ and GFP_Bas_ on which we randomly placed protons on ionizable sites to obtain a 9+ charge state. Because surface side chains may rearrange in response to both the gas-phase conditions and to changes in the charge locations, we discarded all atomic coordinates apart from the Cα and Cβ atoms. At each MC step a proton was moved at random to another residue, after which a short energy minimization was run to let the charges adjust their positions in response to the local potential, comprised of Coulombic interactions, a mean-field solvation at the protein surface, and a short-range repulsion from the backbone defined by the Cα atoms. MC moves were accepted or rejected based on a Metropolis criterion based on the GB’s of protonated sites (Table S1), Coulombic energy, and solvation energy (Figure S8, S9). For each system a total of 10^7^ iterations were performed in 50 replicates. Notably, the lowest energy states were not found in all replicates, but in several of them, indicating that the combined simulation data are sufficient, but that millions of iterations are required, in line with what has been observed for MC schemes with static structures without side chain rearrangements,^48^ underlining the challenge to achieve sufficient sampling for pure and hybrid MC. In all cases the lowest energy states were zwitterionic, with salt bridges forming at the protein surface. We find that the GFP_Bas_ has the lowest Coulombic energy, followed by GFP_WT_ (Table S2). Conversely, the GB contribution is most negative for GFP_Ac_ and least negative for GFP_Bas_, which can be attributed to their different number of D and E residues, as their GBs are the highest among the amino acids. GFP_Bas_ evidently minimizes its total energy by optimizing its charge locations, which is less important for GFP_Ac_ where the low-energy states are dominated by the GBs of protonated side chains. Protonation of high-GB sites does not stabilize the structure *per se*, whereas lowering of the Coulombic energy does, which explains why GFP_Bas_ is more resistant to gas-phase unfolding than the other variants, and why GFP_Ac_ is the most unstable.

### Moving Individual Charged Groups Alters Protein Conformations in the Gas Phase

**Figure 4:**
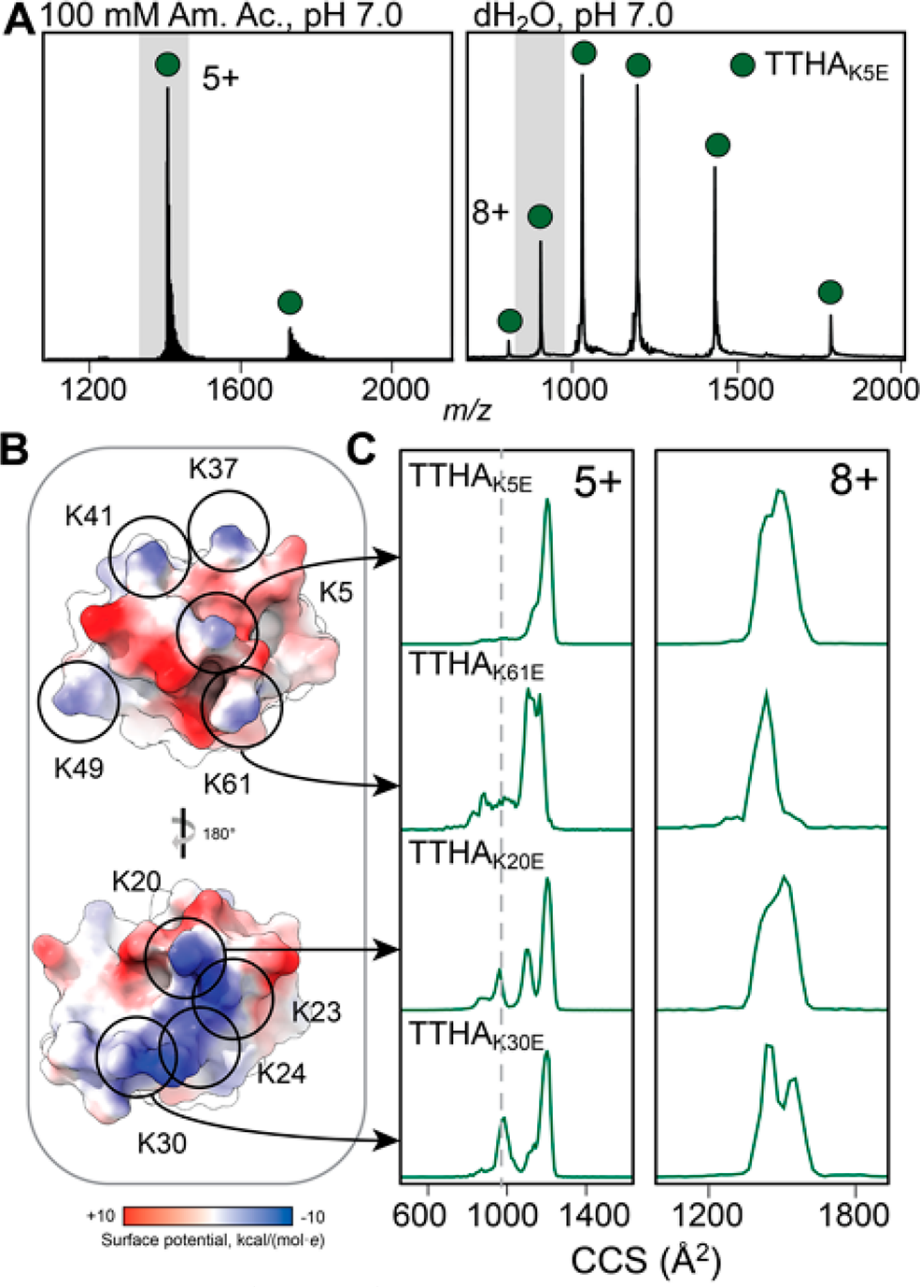
Changing the location of individual lysine residues affects the gas-phase conformation of TTHA1718 variants. (A) ESI mass spectra of TTHA_K5E_ in ammonium acetate show a narrow CSD of 4+ and 5+ ions and in dH_2_O show a broad CSD ranging from 4+ to 9+. (B) The locations of the 9 lysine residues are indicated on the Coulombic surface representation of WT TTHA1718 (PDB ID 2ROE). (C) The CCS distributions of the 5+ charge states of TTHA_K5E_, TTHA_K20E_, TTHA_K30E_, and TTHA_K61E_ sprayed from AmAc at a trap voltage of 5 V show a small native-like population for TTHA_K20E_ and TTHA_K30E_, whereas TTHA_K5E_ and TTHA_K61E_ are mostly unfolded. The 8+ charge states from dH_2_O, which can be considered mostly unfolded, reveal variant-specific differences in their CCS distributions. The dashed line indicates the theoretical CCS of TTHA1718.

The observations from GFP variants raise the question of whether the gas-phase structure of a protein can be affected not just by the number, but also the location of the charges. We thus turned to a third protein system, the small metal-binding protein TTHA1718 from *Thermus thermophilus*, which contains 9 lysines and 1 histidine residue. By exchanging surface lysines at positions 5, 20, 30, or 61 for glutamate, we generated four variants with near-identical structures that differ in the location of one basic and one acidic residue each (TTHA_K5E_, TTHA_K20E_, TTHA_K30E_, and TTHA_K61E_).^49^ Because acidic residues are neutralized during positive-mode ESI, this system enables us to observe the effect of moving a single protonation site on the gas-phase conformation of the protein. To be able to probe a broad range of charge states, we performed MS analysis in ammonium acetate and in dH_2_O which resulted in CSDs ranging from 9+ to 4+ (Figure 4A, S10). The 4+ charge states of the TTHA variants exhibited CCSs of 940–960 Å^2^, in good agreement with the 920 Å^2^ predicted from the NMR structure of the WT protein (Figure S10). The 4+ charge ions were found to unfold readily as the trap voltage was raised above 5 V, and therefore, no CIU traces could be recorded. However, the 5+ charge state showed significant differences between variants: for TTHA_K20E_ and TTHA_K30E_, a small population with a native-like CCS could be detected, as well as more extended states with CCSs of 1100 Å^2^ and 1200 Å^2^, indicating partial unfolding even under the gentlest ESI conditions. TTHA_K5E_ and TTHA_K61E_, on the other hand, showed only an unfolded state with a CCS of approximately 1200 Å^2^ (Figure 4B, C). The lysines are the likely protonation sites due to their high GB. We thus speculated that changing the location of a single lysine residue could change the local Coulombic strain experienced by the protein. However, for the 5+ ion, the number of lysines is greater than the number of ESI charges, meaning multiple charge configurations are still possible. We therefore also considered the higher charge states observed in dH_2_O, the 8+ ion, which should have fully protonated lysines, also exhibited different CCSs (Figure 4C). The CCS values of 1300–1600 Å^2^ are substantially lower than the 2000 Å^2^ expected for a fully extended conformation, indicating that local structural differences can persist even in highly charged ions. We conclude that changing the location of the ionizable sites on the protein surface, and thus the location of charges, affects the gas-phase conformation of protein ions.

## Discussion

Protein charging in positive ESI can be dependent or independent of basic residues: native-like proteins charge according to their SASA regardless of surface charge, whereas the maximum charge for unfolded proteins depends on the number of basic residues.^38,39^ Our results show that droplet charge at the final step of desolvation, which is governed by the Rayleigh limit of droplet stability,^50^ determines the final charge of compact protein ions, not their proton affinity. In the absence of ionizable residues, this charging limit applies even to unfolded proteins: presumably, denatured EXG remains trapped in the ESI droplet and is eventually released in a compact state, giving rise to a native-like CSD, but then easily unfolds in the gas phase due to the absence of a folded protein core. The generation of high charge states commonly associated with unfolded proteins in ESI-MS thus requires ionizable residues which likely drive the expulsion of the protein from the ESI droplet. This interpretation is supported by recent observations from chemically modified proteins.^28^ For native-like proteins, however, a lack of ionizable sites does not appear to alter the conformational landscape. An excess of ionizable side chains, on the other hand, has a direct impact on the conformational stability, as shown for three GFP variants. In solution, GFP_Ac_ and GFP_Bas_ are less stable than GFP_WT_ due to charge repulsion.^43^ In the gas phase, GFP_Bas_, which contains 41 potential protonation sites, displays the highest stability, whereas GFP_WT_ and GFP_Ac_, with 28 and 19 basic residues, respectively, are more susceptible to CIU. Our simulations suggest this stability stems from the scarcity of carboxylate side chains in GFP_Bas_, making the protons distribute over the high number of basic residues for a favorable Coulombic energy. It seems likely that the larger number of basic residues in this variant also allows for a more optimal distribution of charges that minimizes repulsion and creates more opportunities for salt bridges that stabilize compact states in the gas phase.^16,51^ The importance of charge locations is emphasized by the different conformational preferences of TTHA variants in the gas phase. Furthermore, the combination of basic and acidic residues raises the possibility of salt bridge formation on the protein surface, which can also alter gas phase stability.^52^

Together, our findings show that the charge states of native protein ions are governed solely by the Rayleigh limit, whereas the spatial distribution of the charges affects the conformational landscape of the ion. The conclusions have several implications for the use of native MS to study protein folding and interactions:

(1) Ionizable residues are not prerequisite for ESI of intact protein complexes. This finding implies that native MS is suitable for the analysis of integral membrane proteins, despite their uneven surface charge in solution.^33^
(2) The fact that proteins lacking basic residues favor the CRM over CEM reveals the limitations of using charge state signatures to assess the distribution of folded states.^53^ As a result, proteins with few ionizable groups may display diverging charging patterns upon unfolding.
(3) Previous studies have shown that point mutations can affect the CIU pattern of native-like protein ions by modulating their structure in solution.^9,15,54,55^ Our findings reveal that even identically folded proteins can exhibit different CIU fingerprints due to altered surface electrostatics during ESI. Because these differences arise from the locations of ESI charges, not from solution folding, care must be taken when using CIU data to probe solution structures.
(4) Gas-phase unfolding is routinely used to identify compounds that can stabilize protein complexes by occupying specific binding pockets.^11,56−58^ We find that moving charged sites can have a dramatic effect on a protein’s conformation in the gas phase. We speculate that for small proteins with few ionizable sites, such as TTHA, masking a charge site, for example with an ionic ligand, could potentially affect its gas-phase stability. This possibility should be taken into consideration when studying ligand-mediated stabilization of protein complexes in the gas phase.

## Methods

### Protein Preparation

EXG_WT_ and the chargeless mutant EXG_QQQW_ were expressed and purified as described previously, and the chargeless variant subjected to N-terminal acetylation.^34,35^ GFP_WT_, GFP_Ac_, GFP_Bas_, TTHA_K5E_, TTHA_K20E_, TTHA_K30E_, and TTHA_K61E_ were expressed and purified as described.^43,49^ The EXG variants were stored at −80 °C at a concentration of 90 μM and exchanged into 100 mM AmAc, pH 6.9, prior to MS analysis using Biospin 6 columns (BioRad). The TTHA variants were stored at 120 μM in dH_2_O and diluted to 12 μM in dH_2_O for MS analysis. Mass spectra were recorded at concentrations of 15 μM for wild-type and 45 μM for chargeless EXG, and 5 μM for all GFP variants. Horse heart cytochrome C for TWIMS calibration was purchased from Sigma and prepared as described above.

### Native Mass Spectrometry

nESI capillaries were purchased from Thermo. Positive ionization mode mass spectra were acquired on a Micromass LCT ToF modified for analysis of intact protein complexes (MS Vision, The Netherlands) equipped with an offline nanospray source. For the LCT, the capillary voltage was 1.5 kV and the RF lens 1.5 kV. The cone voltage was set to 100 V for normal acquisition and ramped between 50 and 300 V for collisional activation. The pressure in the ion source was maintained at 9.0 mbar. Data were analyzed using MassLynx V4.1 (Waters, UK). Negative ionization mode mass spectra were acquired on an Orbitrap Fusion (Thermo Fisher Scientific, Waltham, MA) equipped with an offline nanoelectrospray source. The instrument was operated in intact protein mode. The capillary voltage was −1.8 kV, the transfer tube temperature was maintained at 40 °C and the pressure in the ion-routing multipole was 0.011 Torr. Collisional activation was performed by increasing the HCD energy in the ion-routing multipole. High-purity nitrogen was used as collision gas. Spectra were recorded using the Orbitrap mass analyzer at a resolution of 60 000 with a high mass mode acquisition window of 1000–5000 *m*/*z* and a scan time of 1 ms. Data were analyzed using Xcalibur 3.0 (Thermo Scientific, Waltham, MA).

### Ion Mobility Mass Spectrometry of EXG and TTHA1718

IMMS data for EXG and TTHA1718 were recorded on a Waters Synapt G1 TWIMS MS modified for analysis of intact protein complexes (MS Vision, The Netherlands) and equipped with an offline nanospray source. The capillary voltage was 1.5 kV. The cone voltage was set to 10 V, and the source temperature was maintained at 20 °C. The source pressure was adjusted to 8 mbar. The ion trap voltage was ramped from 5 to 25 V for collisional activation, transfer voltage was 10 V. IM settings were: Wave height 12 V, wave velocity 450 m/s for EXG, and wave height 10 V, wave velocity 250 m/s for TTHA1718. IMS gas was nitrogen with a flow of 15 mL/min and collision gas argon with a flow of 4 mL/min. Horse heart cytochrome C and human insulin were used for TWIMS calibration for EXG and TTHA1718, respectively.

### Ion Mobility Mass Spectrometry of GFP

IMMS measurements of equimolar mixtures of GFP_WT_ with either GFP_Ac_ or GFP_Bas_ were performed on a Waters Synapt G1 ion mobility mass spectrometer equipped with a linear field drift tube to facilitate direct CCS determination, and an offline nanospray source. Protein samples were introduced using in-house produced gold-coated borosilicate capillaries. The pressure in the source region was maintained at 5.0 mbar. Mass spectra were recorded at drift voltages between 40 and 120 V at an ion trap voltage of 20 V for CCS determination. For collisional unfolding measurements, the ion trap voltage was ramped from 10 to 100 V in 5 V increments. The drift gas was helium and collision gas was argon. Stabilities of the native-like states of states GFP_Bas_ or GFP_Ac_ were calculated relative to GFP_WT_, measuring either GFP_Bas_ or GFP_WT_ with GFP_Ac_ as an internal standard in the same MS experiment, from three independent repeats. Importantly, we observed the same CSDs on all four MS platforms when using the same solution conditions. Theoretical CCS values of EXG (PDB 6QFS) and GFP (PDB 2B3P as well as Rosetta structures of the GFP_Ac_ and GFP_Bas_ variants) were calculated using IMPACT using the PA method and an empirical correction factor of 1.14.^59,60^ All IMMS data were analyzed using the PULSAR software package.^10^

## Supporting Information Available

Experimental details for computational simulations; protein sequences; supplementary tables summarizing energy terms for MC (ST1) and total energies (ST2); and supplementary figures showing the structure of EXG (S1), salt adduction to EXG (S2), charge reduction of EXG (S3), CCS distributions of EXG (S4), CIU profiles of EXG (S5) and GFP (S6), quantification of GFP stability (S7), a schematic of the MD strategy (S8), solvation potentials (S9), and mass spectra of TTHA variants (S10) (PDF)

## Abbreviations

ESI-MS: electrospray ionization-mass spectrometry
SASA: solvent-accessible surface area
CRM: charged residue model
CEM: chain ejection model
AmAc: ammonium acetate
CSD: charge state distribution
CCS: collision cross section
CIU: collision-induced unfolding
TMAO: trimethylammonia-*N*-oxide
