# Supplementary material for: Charge Engineering Reveals the Roles of Ionizable Side Chains in Electrospray Ionization Mass Spectrometry
Source: JACS Au. 2021 Nov 29;1(12):2385–93. doi: 10.1021/jacsau.1c00458 (PMC8717373; doi:10.1021/jacsau.1c00458)
Supplement: Supplementary file 1 — au1c00458_si_001.pdf [file au1c00458_si_001.pdf]

Supplementary Information to

Charge engineering reveals the roles of ionizable side chains in electrospray ionization mass spectrometry

Mia L. Abramsson,[1‡] Cagla Sahin,[1,2‡] Jonathan T. S. Hopper,[3‡] Rui M. M. Branca,[4] Jens Danielsson,[5] Mingming Xu,[5] Shane A. Chandler,[3] Nicklas Österlund,[5] Leopold L. Ilag,[6] Axel Leppert,[7] Joana Costeira-Paulo,[8] Lisa Lang,[5] Kaare Teilum,[2] Arthur Laganowsky,[9] Justin L. P. Benesch,[3] Mikael Oliveberg,[5] Carol V. Robinson,[3] Erik G. Marklund,[8,*] Timothy M. Allison,[11,*] Jakob R. Winther,[2] and Michael Landreh[1,*]

[1] Department of Microbiology, Tumor and Cell Biology, Karolinska Institutet, Solnavägen 9, 171 65 Stockholm, Sweden [2] Linderstrøm-Lang Centre for Protein Science, Department of Biology, University of Copenhagen, Ole Maaløes vej 5, 2200 Copenhagen, Denmark [3] Department of Chemistry, University of Oxford, South Parks Road, Oxford OX1 3QZ, UK [4] Department of Oncology-Pathology, Science for Life Laboratory and Karolinska Institutet, Stockholm, Sweden. [5] Department of Biochemistry and Biophysics, Stockholm University, 106 91 Stockholm, Sweden [6] Department of Material and Environmental Chemistry, Stockholm University, 106 91 Stockholm, Sweden [7] Department of Biosciences and Nutrition, Karolinska Institutet, Neo, 141 83 Huddinge, Sweden [8] Department of Chemistry - BMC, Uppsala University, Box 576, 751 23, Uppsala, Sweden [9] Department of Chemistry, Texas A&M University, College Station, TX 77843, USA [10] Biomolecular Interaction Centre, School of Physical and Chemical Sciences, University of Canterbury, Christchurch 8140, New Zealand

Methods

CD spectroscopy. Circular dichroism (CD) spectra were recorded on a Chirascan CD spectrometer (Applied Photophysics, UK) using a quartz cuvette with an optical path length of 2 mm. Acquisitions were made in the spectral region of 190–250 nm with a step size of 1 nm and a sampling time of 4 s per data point. Measurements were made on samples of 20 μM EXG_{WT} or EXG_{QQQW} in 10 mM NaPi, pH 7.3, or 50% acetonitrile in H_2O, at 293 K. Spectra were blank-subtracted and cut off above a HT voltage of 700.

Prediction of z_{max} and z_{avg}

For calculation of z_{max}, which describes the maximum charge according to the Rayleigh limit, the strategy of Fernandez de la Mora was used [1]. For a droplet with the same molecular weight as the protein, a spherical shape, a density of 1 g/cm^3, and the surface tension of water (0.072 N/m) were assumed. For calculation of z_{avg}, we employed the data for 19 native-like protein complexes reported by Kaltashov and Mohimen [2]. Plotting the average charge as a function of surface area yields a correlation of $z_{avg} = -0.0087\,(SASA)^2 + 1.939$, with an R^2 value of 0.9755. SASAs for all proteins in this study were calculated using UCSF Chimera [3].

Metropolis Monte Carlo Simulations

The crystal structure of the superfolder GFP (pdb code 2B3P [4]) was used as a starting point for the wild-type protein, and Rosetta structures were used for the basic and acidic variants

[5]. To model the proteins in the MS experiments, which were purified using a His-tag, the three structures were complemented with a sequence of six histidines following one N-terminal Met and a Gly redidue. The His-tails were modelled using PyMol to stick out straight from the rest of the structure in a beta-strand conformation, under the assumption that the tail can pick up charges and reduce the Coulombic repulsion by protruding in a straight conformation.

The His-tagged GFP structures were used as input for the Monte Carlo (MC) simulations. It is known that side chains can rearrange in response to the gas-phase condition [6], but it is also reasonable to assume that side chains and other chemical groups will move to solvate charged groups, form salt bridges when favourable, etc. The finer details in crystallographic structures therefore risk being invalid for gas-phase proteins, or at least act to create a bias towards charge locations that are present in the crystal. We therefore took a residue-level coarse-grained approach, where charges are moved around between ionizable sites in a Metropolis MC scheme and allowed to relax in a short energy minimization at each step. The energy of any state is defined by three components: a) The electrostatic interaction between charges, b) the solvation energy of charges at the protein surface, and c) the gas-phase basicity of protonated groups.

In the coarse-graining we discard most atomic coordinates in the input structure, only keeping the C_α atoms, the C_β atoms of ionizable residues (His, Lys, Arg, Glu, Asp) as well as the N-atom in the N-terminal amine and the C-atom in the C-terminal carboxylic group. In our model the C_β and the terminal N- and C-atoms serve as anchor points for charges that are free to move within a distance from the anchor they are attached to, where the distance is determined by the number of covalent bonds in the sidechain from the charged group to the C_β. More precisely, the maximum allowed distance d is defined as follows:

$$d = \begin{cases} d_{C-C}, & n = 1 \\ n\, d_{C-C}\, \sin\left(\frac{109.5}{2}\right), & n > 1 \end{cases}$$

where $d_{C\text{-}C}$ = 1.54 Å is the distance of a C-C bond, n the number of bonds separating the charge from the anchor point, and the sinus function reflecting the tetrahedral geometry of the bonds around the C-atoms. See Table S1 for the values of n for the different types of ionizable

sites. To prevent opposite charges from coming unphysically close to each other, we imposed a minimum distance $d_{\min}$ = 3 Å between charges, reflecting a an approximate N-O distance in salt bridges between amino acid side chains [7].

Coulombic interactions between charged sites were calculated using Coulomb's law. A relative dielectric constant of 2.0 was used to reflect a compromise between the presence of the protein and the vacuum surrounding and is therefore low compared to values commonly employed for continuum electrostatics in protein. Schnier et al.[8] reported that a dielectric constant of 2.0 ± 0.2 makes the best match for experimental observations of charging in cytochrome C. While GFP is larger than cytochrome C, a charge near the GFP surface will not experience a much larger protein dielectric than on cytochrome C, hence we are confident that a value of 2.0 will yield qualitatively sound results. It should be noted however that the calculations might be sensitive to the choice of dielectric constant and other parameters, and the exact quantitative results must therefore be treated with appropriate caution, but importantly that does not preclude a qualitative analysis of trends observed using a reasonable parameter set.

To prevent the mobile charges from penetrating the protein and form salt bridges through the backbone, we defined a physical barrier using the C_α atoms. A repulsive potential was defined around each C_α atom according to $U_r = k_r(d_r - r)^2$, where k_r = 250 kJ/(mol Å^2) is a force constant, d_r = 3.5 Å is the distance where the repulsion starts, and r is the distance between a charge and the C_α atom.

The charge solvation at the protein surface, representing the rearrangement of local dipoles etc, was modelled with a potential that switches from zero at far distances to U_{sol} at shorter distances, where a cosine function is used as a switching function. More specifically,

$$U_{sol} = -0.5\, E_{\min} \left[1 - \cos\left(180 \frac{d_{sol} - r}{d_{sol} - d_r}\right)\right]$$

where d_{sol} = 5.5 Å is the distance where the switching starts, d_r is where the repulsion starts (see above), and $E_{\min}$ is the energy minimum for this potential. U_{sol} as defined above is only evaluated on the interval $d_{sol} < r \leq d_r$, and U_{sol} is stipulated to be equal to zero and $E_{\min}$ at

longer and shorter distances, respectively. Although the solvation energy can be assumed to vary across the protein surface [9], we take an agnostic approach and set $E_{\min}$ to a mean-field value of 62 760 J/mol that has been used before in the literature [10]. In contrast to all other contributions to the total potential, only the interaction with the closest C_α is used to calculate the solvation energy, which prevents multiple inclusions of this energy term for a given charge carrier. The interaction between a charge and the C_α from the same residue requires special treatment however because in that case there is no complete side chain blocking access to the backbone. The limits and reference distances used to calculate such self-interaction was therefore decreased by 2.5 Å for both U_r and U_{sol}. The repulsion and the solvation potentials are illustrated in Fig S9.

The last contribution to the energy comes from the GB. For each site that is protonated a contribution to the energy is made corresponding to the intrinsic GB for that site. GB values used in the simulations are shown in table S1.

Some internal ionizable residues can be assumed to have a constant protonation state throughout the simulations. We inspected the structures to determine which residues were to be given constant charge based on literature and interactions with surrounding residues. Arg96 and Glu222 (residue numbering corresponding to the 2B3P structure) flanking the chromophore were both assumed to be charged. His169 and His181 in contrast was given a constant zero charge.

MC simulations were carried out for the three systems. Initial protonation states were generated by randomly placing protons on the ionizable sites until the net charge was 9+. At each MC step a trial move is made, where one protonated and one deprotonated site is chosen randomly to get deprotonated and protonated, respectively. A steepest descent energy minimization is then run to let the charged sites adjust according to the potential terms described above, whereupon the total energy is calculated. The move is accepted or rejected based on a Metropolis criterion, assuming a temperature of 300 K. 40 000 iterations were carried out in this manner, after which the simulation was reset with a new randomized proton configuration. The protonation states were reset in this way 50 times per simulation, and the results were pooled from 50 replicate simulations, yielding a total of 10 000 000

iterations per system. After each randomization of the starting proton configuration an extra 100 iterations were done to allow for initial equilibration. We note that even though each simulation comprised 2000 000 iterations, the state with lowest energy were found in many, but not all, simulations. Pooling the results from multiple simulations overcame this sampling issue. This observation indicates a need for millions of iterations in computations akin to ours for convergence on the correct result. Since our coarse-grained model most likely leads to a smoother energy landscape than in fully atomistic models, it is not unlikely that even more iterations are needed for more detailed representations.

The absolute probability p of a state cannot, strictly speaking, be calculated without knowledge of the partition function. The relative probabilities p' of the states can however be exactly determined using $p' = e^{-E/k_BT}$, where E is the energy of the given state. We note that in our model all states are equally degenerate, meaning that no entropic differences need to be considered, , and that we are not dependent on extensive sampling of the individual states but can get their Boltzmann weights even if they are only visited once during the simulation. If we assume that all relevant states and their relative probabilities have been found, we can obtain the absolute probabilities by normalizing the relative probabilities so that their sum equals unity, that is, $p = Ap'$, where $A = 1/\sum p'$. Our large number of simulations and replicas seems to allow us to make that assumption, since several of the replicas return the same lowest-energy states out of an extremely large number of possible states. Knowing the absolute probabilities, we can calculate the ensemble averages of a quantity X via $\langle X \rangle = \sum p_i X_i$, where p_i and X_i are the probability and value of X for state i. The ensemble averages for the total energy (E_{tot}), the GB-contribution to the energy (E_{GB}), the Coulombic energy (E_{Coul}), and the solvation energy (E_{sol}) for the three GFP systems are listed in Table S2.

Protein sequences

EXG$_{WT}$

Arg: 1 Lys: 1 Asp: 1 Glu: 0

ASSGPAGCQVLWGVNQWNTGFTANVTVKNTSSAPVDGWTLTFSFPSGQQVTQAWS
STVTQSGSAVTVRNAPWNGSIPAGGTAQFGFNGSHTGTNAAPTAFSLNGTPCTVG

EXG$_{QQQW}$

Arg: 0 Lys: 0 Asp: 0 Glu: 0

ASSGPAGCQVLWGVNQWNTGFTANVTVQNTSSAPVQGWTLTFSFPSGQQVTQAWS
STVTQSGSAVTVQNAPWNGSIPAGGTAQFGFNGSWTGTNAAPTAFSLNGTPCTVG

GFP$_{WT}$

Arg: 8 Lys: 20 Asp: 18 Glu: 16

MGHHHHHHGGASKGEELFTGVVPILVELDGDVNGHKFSVRGEGEGDATNGKLTLKFIC
TTGKLPVPWPTLVTTLTYGVQCFSRYPDHMKRHDFFKSAMPEGYVQERTISFKDDGTY
KTRAEVKFEGDTLVNRIELKGIDFKEDGNILGHKLEYNFNSHNVYITADKQKNGIKAN
FKIRHNVEDGSVQLADHYQQNTPIGDGPVLLPDNHYLSTQSVLSKDPNEKRDHMVLLE
FVTAAGITHGMDELYK

GFP$_{Ac}$:

Arg: 6 Lys: 13 Asp: 25 Glu: 24

MGHHHHHHGGASKGEELFDGVVPILVELDGDVNGHEFSVRGEGEGDATEGELTLKFIC
TTGELPVPWPTLVTTLTYGVQCFSDYPDHMDQHDFFKSAMPEGYVQERTISFKDDGTY
KTRAEVKFEGDTLVNRIELKGIDFKEDGNILGHKLEYNFNSHDVYITADKQENGIKAE
FEIRHNVEDGSVQLADHYQQNTPIGDGPVLLPDDHYLSTESALSKDPNEDRDHMVLLE
FVTAAGIDHGMDELYK

GFP$_{Bas}$

Arg: 16 Lys: 25 Asp: 12 Glu: 14

MGHHHHHHGGASKGERLFTGVVPILVELDGDVNGHKFSVRGEGEGDATRGKLTLKFIC
TTGKLPVPWPTLVTTLTYGVQCFSRYPKHMKRHDFFKSAMPEGYVQERTISFKKDGTY
KTRAEVKFEGRTLVNRIELKGRDFKEKGNILGHKLEYNFNSHNVYITADKRKNGIKAN
FKIRHNVKDGSVQLADHYQQNTPIGRGPVLLPRNHYLSTRSALSKDPKEKRDHMVLLE
FVTAAGITHGMDELYK

TTHA$_{K5E}$

Arg: 0 Lys: 8 Asp: 1 Glu: 11

MLKLEVEGMTSNHSVMAVTKALKKVPGVEKVEVSLEKGEALVEGTADPKALVQAVEEEGYKAEVLA

TTHA$_{K20E}$

Arg: 0 Lys: 8 Asp: 1 Glu: 11

MLKLKVEGMTSNHSVMAVTEALKKVPGVEKVEVSLEKGEALVEGTADPKALVQAVEEEGYKAEVLA

TTHA$_{K30E}$

Arg: 0 Lys: 8 Asp: 1 Glu: 11

MLKLKVEGMTSNHSVMAVTKALKKVPGVEEVEVSLEKGEALVEGTADPKALVQAVEEEGYKAEVLA

TTHA$_{K61E}$

Arg: 0 Lys: 8 Asp: 1 Glu: 11

MLKLKVEGMTSNHSVMAVTKALKKVPGVEKVEVSLEKGEALVEGTADPKALVQAVEEEGYEAEVLA

Supplementary References

[1] J. F. De La Mora, *Anal. Chim. Acta* **2000**, *406*, 93–104.

[2] I. A. Kaltashov, A. Mohimen, *Anal Chem* **2005**, *77*, 5370–5379.

[3] E. F. Pettersen, T. D. Goddard, C. C. Huang, G. S. Couch, D. M. Greenblatt, E. C. Meng, T. E. Ferrin, *J Comput Chem* **2004**, *25*, 1605–1612.

[4] J. D. Pédelacq, S. Cabantous, T. Tran, T. C. Terwilliger, G. S. Waldo, *Nat. Biotechnol.* **2006**, *24*, 79–88.

[5] M. S. Lawrence, K. J. Phillips, D. R. Liu, *J. Am. Chem. Soc.* **2007**, *129*, 10110–10112.

[6] D. Van Der Spoel, E. G. Marklund, D. S. D. Larsson, C. Caleman, *Macromol. Biosci.* **2011**, *11*, 50–59.

[7] K. T. Debiec, A. M. Gronenborn, L. T. Chong, *J. Phys. Chem. B* **2014**, *118*, 6561–6569.

[8] P. D. Schnier, D. S. Gross, E. R. Williams, *J. Am. Chem. Soc.* **1995**, *117*, 6747–6757.

[9] M. Miteva, P. A. Demirev, A. D. Karshikoff, *J. Phys. Chem. B* **1997**, *101*, 9645–9650.

[10] P. D. Schnier, D. S. Gross, E. R. Williams, *J. Am. Soc. Mass Spectrom.* **1995**, *6*, 1086–1097.

[11] R. Marchese, R. Grandori, P. Carloni, S. Raugei, *PLoS Comput. Biol.* **2010**, *6*, e1000775.

Supplementary Tables

Table S1. Number of bonds from anchor point and intrinsic GB for ionizable side chains and termini. Values for side chains were taken from Marchese *et al* [11]. The N-terminal amine was given the same GB as the Lys side chain, and the C-terminal carboxylate was given a GB equal to the average of the GBs of Asp and Glu side chains.

Residue/terminus	*n*	GB (kJ/mol)
Arg	2	1026
Lys	2	978
His	2	971
Asp	2	1328
Glu	3	1356
N-terminus	0	978
C-terminus	2	1342

Table S2. Ensemble averages from the MC simulations.

System	$\langle E_{tot} \rangle$ (kJ/mol)	$\langle E_{GB} \rangle$ (kJ/mol)	$\langle E_{Coul} \rangle$ (kJ/mol)	$\langle E_{sol} \rangle$ (kJ/mol)
GFP_{WT}	-52 540	-47 205	-3 138	-2 197
GFP_{Bas}	-51 550	-44 579	-4 213	-2 758
GFP_{Ac}	-69 833	-64 797	-2 967	-2 069

Supplementary Figures

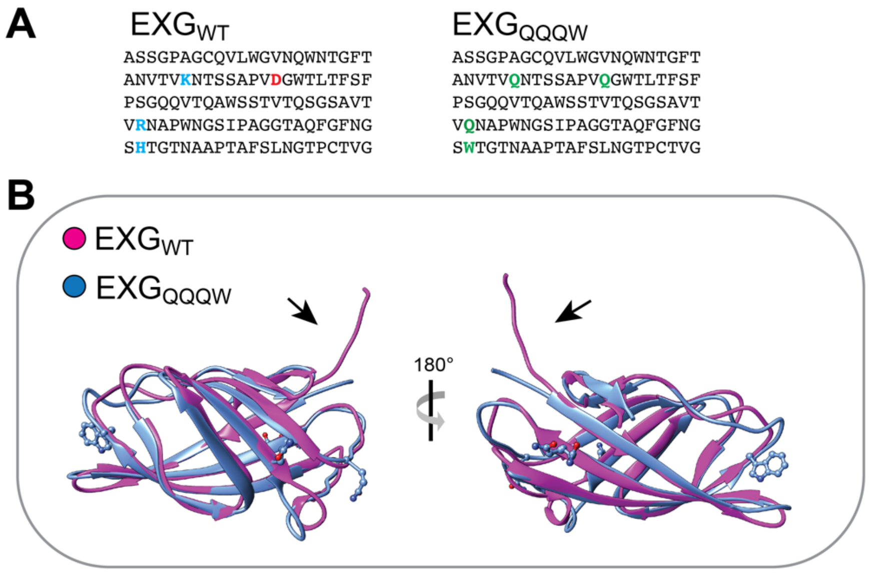

Figure S1. (A) Sequences of EXG_{WT} and EXG_{QQQW}. Basic and acidic residues in EXG_{WT} are highlighted in blue and red, and the chargeless replacements in EXG_{QQQW} in green. (B) Overlay of the high-resolution structures of EXG_{WT} (PDB ID 1EXG) and EXG_{QQQW} (PDB ID 6QFS). The four charge replacement residues are shown as ball and stick rendering. The six disordered N-terminal residues of EXG_{WT} (arrow) are absent from the crystal structure of EXG_{QQQW}.

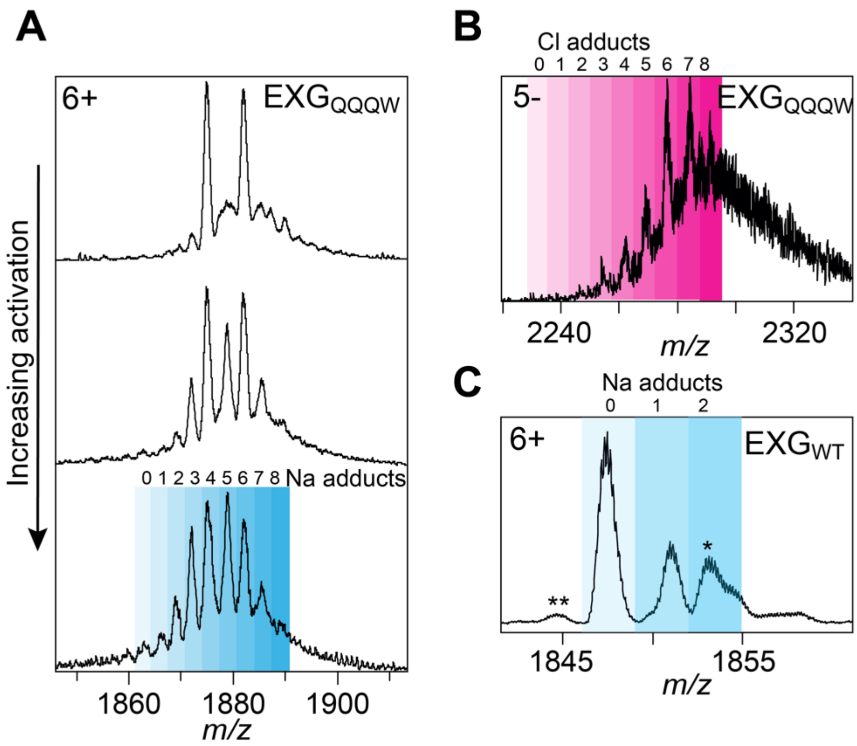

Figure S2. (A) The main charge state of EXG$_{QQQW}$ contains two populations with higher masses than expected. Collisional activation reveals a broad Na+ adduct distribution reaching the expected protein mass. (B) Ionization of EXG$_{QQQW}$ in negative polarity shows multiple Cl- adducts. (C) Using the same conditions as in (A), EXG$_{WT}$ displays the expected mass. One and two asterisks indicate a deamidation (-17 Da) and a potassium adduct (+39 Da), respectively.

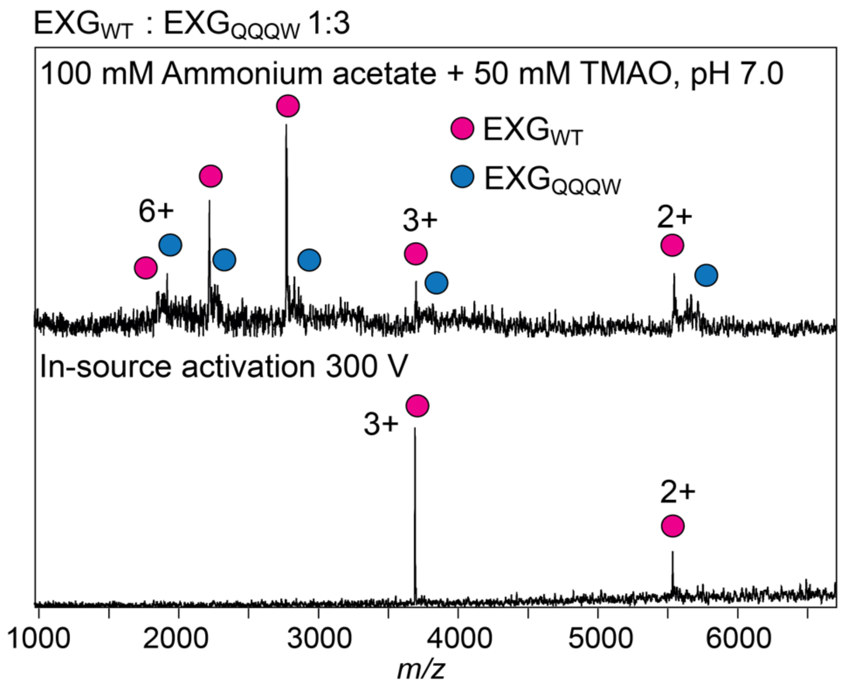

Figure S3. Modulating the CSDs of wild-type and charge-depleted EXG. Addition of TMAO resulted in moderate charge reduction for EXG_{WT} and significant loss of EXG_{QQQW} ion signal (top). At 300 V, EXG_{WT} could be detected with an average charge of 2.7, while EXG_{QQQW} signal was lost completely (bottom).

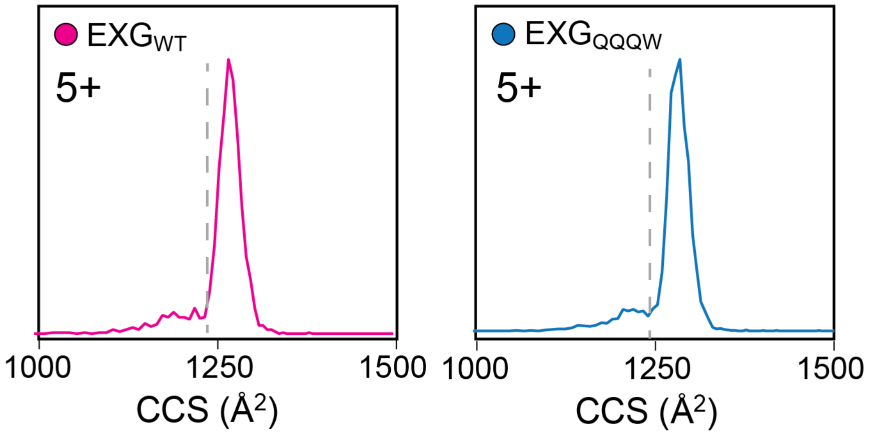

Figure S4. CCS distributions for the 5+ charge states of EXG_{WT} and EXG_{QQQW} show good agreement with the values computed from the crystal structure (dashed lines).

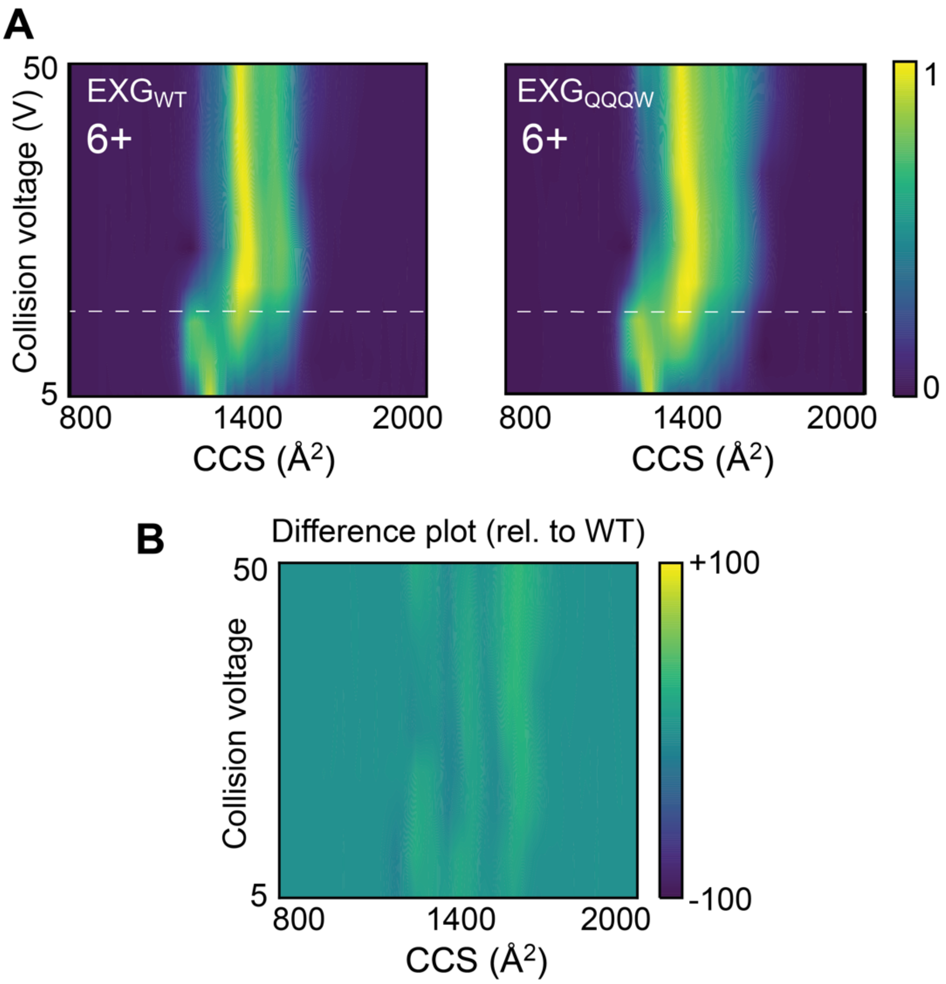

Figure S5. (A) CIU profiles of the 6+ charge states of EXG_{WT} (left) and EXG_{QQQW} (right) under native conditions show virtually identical unfolding steps, with the compact state unfolding at around 15V collision energy (dashed lines). (B) The calculated CIU difference plot of the chargeless variant relative to the WT shows no significant differences in gas-phase stability. Computing the RMSD value between unfolding plots of EXG_{WT} and EXG_{QQQW}, yields a value of ~5.9%. For comparison, the RMSD values between replicate plots for the same protein are around ~3-4%.

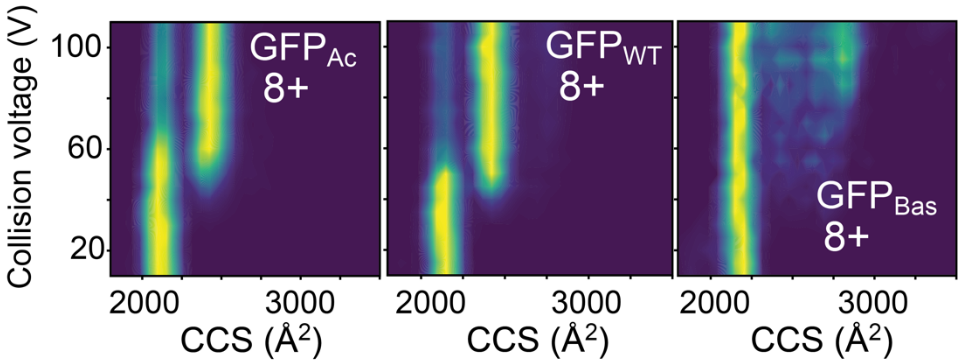

Figure S6. CIU plots of the 8+ charge states of GFP$_{Ac}$, GFP$_{WT}$, and GFP$_{Bas}$ show that the basic variant is notably more resistant to unfolding than WT and acidic variant.

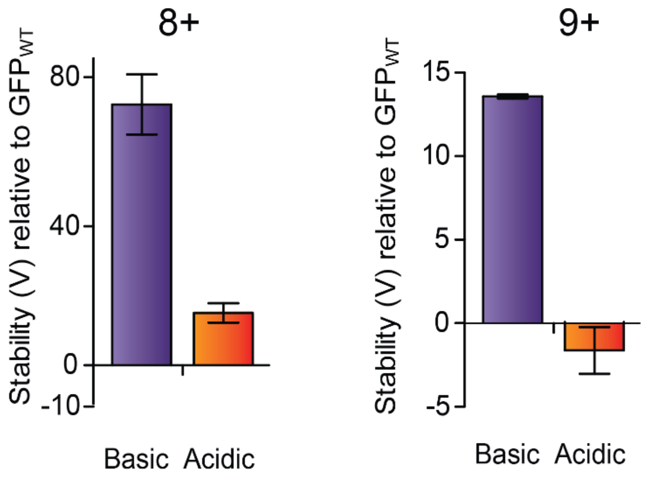

Figure S7. Quantification of the charge-induced stabilization of the native-like states of GFP$_{Ac}$ and GFP$_{Bas}$. To minimize variability between experiments associated with differences in lab conditions, the GFP data were collected in pairs within the same spectra: WT and +15, and +15 and -30, taking advantage of the resolvable difference in mass between the chosen pairs of proteins. The relative stability of the native-like form for the +15 and -30 variants to the WT protein were then calculated. Stabilization is expressed in V required to unfold the most compact state, relative to GFP$_{WT}$. Error bars indicate standard deviations from three separate experiments.

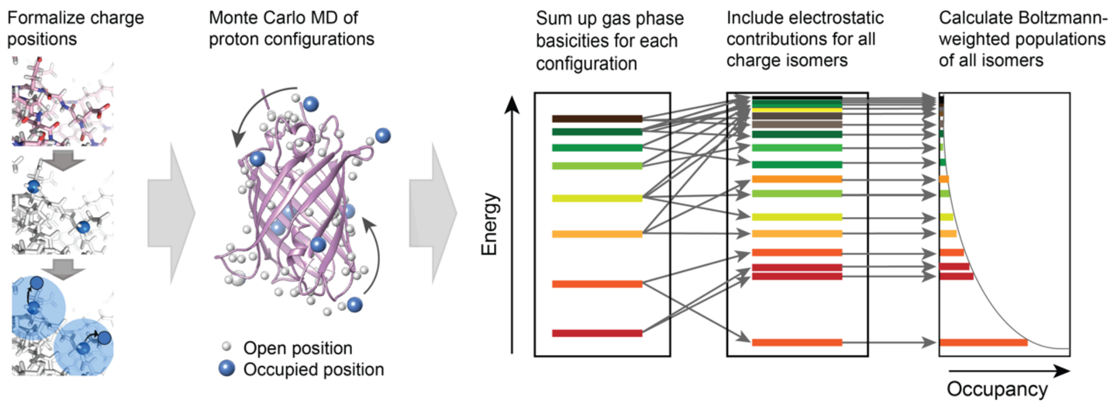

Figure S8. Strategy for the prediction of preferred charge location on GFP_{Ac}, GFP_{WT}, and GFP_{Bas}.

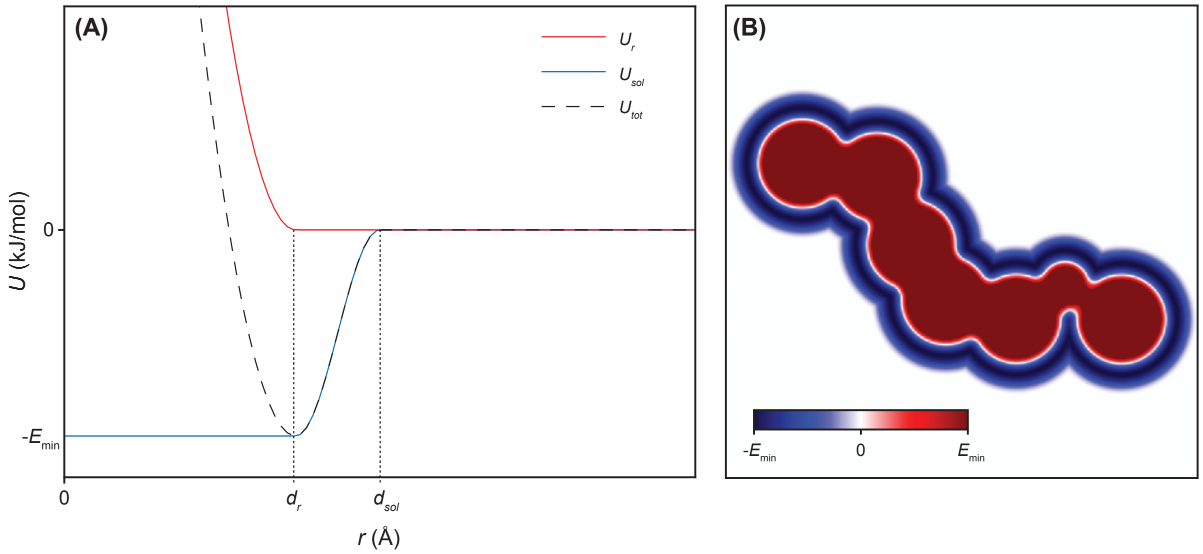

Figure S9. Repulsion and solvation potentials for a charge near the protein surface. (A) The potential terms as functions of distance between the charge and C_α atom. (B) Illustration of the potential near a chain of seven C_α atoms in two dimensions. Note that one of the C_α atoms have a smaller apparent radius, reflecting that it belongs to the residue that carries the charge in question.

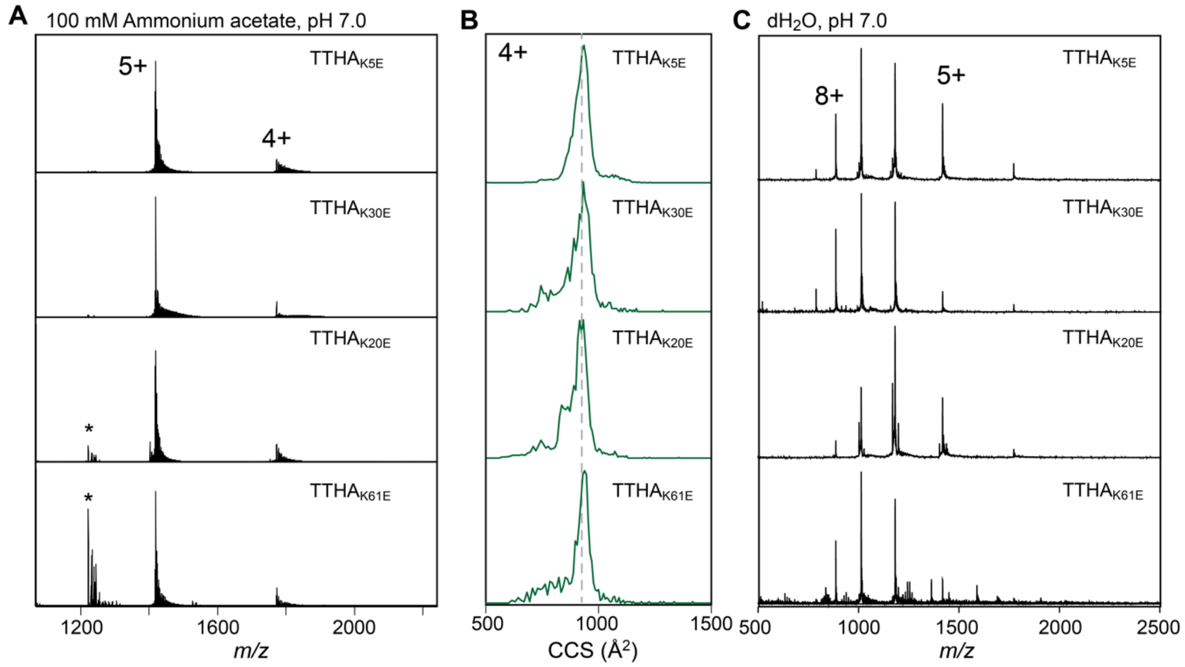

Figure S10. (A) ESI-mass spectra of TTHA variants in 100 mM AmAc show a narrow CSD expected for a folded protein. Asterisks indicate interference from residual salt. (B) CCS distributions for the 4+ charge states show a major population with native-like CCS for all variants. (C) ESI-MS in dH_2O reveals broad charge state distributions ranging from 4+ to 9+ for $TTHA_{K5E}$ and $TTHA_{K61E}$, and 4+ to 8+ for $TTHA_{K20E}$ and $TTHA_{K30E}$.
